# Measurement of tissue optical properties in the 400 to 700 nm range to assess light penetration depths for laser treatment of upper tract urothelial carcinomas

**DOI:** 10.1117/1.JBO.29.12.125001

**Published:** 2024-12-10

**Authors:** Himemi Watabe, Yu Shimojo, Asako Shingu, Hidenori Ito, Hideo Fukuhara, Makito Miyake, Keiji Inoue, Kiyohide Fujimoto, Takahiro Nishimura

**Affiliations:** a Osaka University, Graduate School of Engineering, Osaka, Japan; b Osaka Metropolitan University, Graduate School of Medicine, Osaka, Japan; c Research Fellow of Japan Society for the Promotion of Science, Tokyo, Japan; d SBI Pharmaceuticals Co., Ltd., Tokyo, Japan; e Kochi Medical School, Department of Urology, Kochi, Japan; f Nara Medical University, Department of Urology, Nara, Japan

**Keywords:** upper urinary tract, optical properties, light penetration depth, human tissue, laser treatment, upper tract urothelial carcinoma

## Abstract

**Significance:**

For therapeutic approaches for upper tract urothelial carcinomas, the absorption $\mu_{a}$ and reduced scattering $\mu_{s}^{\prime }$ coefficients of these tissues are essential parameters to quantitatively evaluate the distribution of light treatment effects.

**Aim:**

The $\mu_{a}$ and $\mu_{s}^{\prime }$ spectra of the human ureter, fatty tissue, ureteral and renal pelvic carcinomas, and porcine ureter and fatty tissue are measured over 400 to 700 nm to evaluate projected light penetration depths δ.

**Approach:**

The optical properties were determined with a double integrating sphere optical system and inverse Monte Carlo methods. δ was calculated and compared between normal and cancerous human tissues as well as between normal human and porcine tissues.

**Results:**

$\mu_{a}$ and $\mu_{s}^{\prime }$ spectra of each tissue were determined. The δ of the normal human ureter was less than those of the ureteral and renal pelvic carcinomas, whereas that of the porcine ureter was similar to that of the human ureter over 400 to 600 nm and ∼0.2 times smaller above 600 nm.

**Conclusion:**

Optical properties of human and porcine upper urinary tracts provide insights into light distributions and the validity of *ex vivo* porcine models in preclinical evaluations of laser treatments.

## 1 Introduction

Upper tract urothelial carcinoma (UTUC) is a malignant tumor occurring in the urothelial mucosa of the ureter and renal pelvis. Radical nephroureterectomy has been considered the standard of care.^1^^,^^2^ This procedure has been overused for low-grade tumors with little risk of metastatic progression, and even for some early-stage high-grade superficial tumors,^3^^,^^4^ leading to increased interest in organ-sparing strategies to preserve renal function. One of the most promising strategies is an endoscopic approach using laser light that can be delivered minimally invasively via an optical fiber and can interact specifically with tumors.^5^ These modalities include photodynamic therapy (PDT)^6^ and laser ablation (LA).^7^ Laser-tissue interactions in these treatments are related to optical absorption and scattering by the tumors, making information on the spatial distribution of light in the tissue crucial for estimating treatment effects.^8^

The light distribution in the tissue can be analyzed using absorption ($\mu_{a}$) and reduced scattering ($\mu_{s}^{\prime }$) coefficients, which vary with wavelength and tissue structure.^9^ The accuracy of these parameters affects the light distribution analysis for the tissue. The structure of the upper urinary tract tissue primarily comprises the ureter and fatty tissue.^10^ UTUC optical attenuation coefficients have been measured via optical coherence tomography.^11^^,^^12^ However, because these measurements were limited to the light source wavelengths (1300 nm), they cannot be applied to the evaluation of wavelengths used in endoscopic laser treatments, such as 410, 630, 635, 664, and 690 nm for PDT and 450 and 532 nm for LA. It is thus necessary to acquire the $\mu_{a}$ and $\mu_{s}^{\prime }$ spectra of human upper urinary tract tissues to understand light distributions for evaluating safety and efficacy in laser treatments.

Here, we measured $\mu_{a}$ and $\mu_{s}^{\prime }$ spectra in the visible wavelength range for human ureter, fatty tissue, and ureteral and renal pelvic carcinomas to quantitatively evaluate projected light penetration depths (δ) in each tissue. The measurements were performed with a double integrating sphere (DIS) optical system (the gold standard for optical properties) and inverse Monte Carlo (IMC) calculations.^13^^–^^16^ A DIS enables simultaneous measurements of diffuse reflectance ($R_{d}$) and total transmittance ($T_{t}$), which reduces sample degradation during measurements. By comparing the measured values of $R_{d}$ and $T_{t}$ with the IMC values, the $\mu_{a}$ and $\mu_{s}^{\prime }$ parameters for normal and cancerous human upper urinary tract tissues are determined. Furthermore, because the optical properties of porcine models are likely to be used in preclinical evaluations, they are also measured for comparison with human tissue. Subsequently, δ is calculated from the values of $\mu_{a}$ and $\mu_{s}^{\prime }$, to investigate differences in light distributions across various tissues of human and porcine upper urinary tracts. These results will provide a quantitative understanding of light distributions in human upper urinary tracts and enable the development of analytical models and *ex vivo* tests for selecting appropriate wavelengths for UTUC laser treatments.

## 2 Materials and Methods

### 2.1 Sample Preparation

**Fig. 1:**
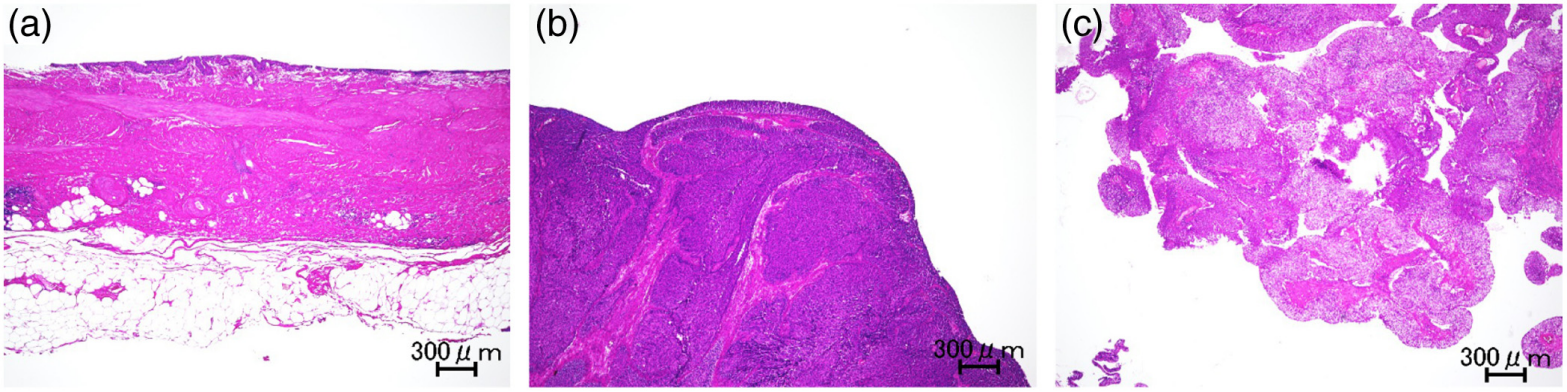
Hematoxylin and eosin stained images of (a) normal ureter, (b) ureteral carcinoma, and (c) renal pelvic carcinoma. Scale bars: 300  μm.

Normal and cancerous human tissues were obtained from surgeries performed at Kochi Medical School and Nara Medical University. A study protocol approved by the Research Ethics Committee of Osaka University (approval number: L031) was followed, and informed consent forms were signed by participating patients before surgery. Six Asian patients (four males and two females) aged 60 to 82 (73±8) years old were enrolled. Measurements were performed within 6 h after surgery. The excised tissues were preserved in saline-moistened gauze until measurements were performed. From the excised ureters and renal pelvises, normal ureter, ureteral carcinoma, and renal pelvic carcinoma samples were cut into 1 cm cubes. Figure 1 shows the representative hematoxylin and eosin staining images of a normal ureter, a ureteral carcinoma, and a renal pelvic carcinoma. The normal ureter was composed of the mucosal epithelium, connective tissue, muscle tissue, and peripheral fatty tissue, and the peripheral fatty tissue had adipocytes with fewer extracellular matrix than the upper layers. In the cancerous tissues, malignant epithelial cells proliferated. Fatty tissue around the ureter was separated using a scalpel. Fatty tissue and cancerous tissues were trimmed with scissors to adjust the sample thicknesses. Each sample thickness was measured three times using a micrometer (MDC-25PX, Mitsutoyo), and the average value was used. Samples were sandwiched between glass slides using multiple stainless-steel spacers with thicknesses of 0.1, 0.5, and 1.0 mm to maintain the thickness without compression. To prevent drying, 1 to 2 drops of saline were added to the tissues, which were then sealed with plastic paraffin film. For the porcine samples, tissues were purchased from Tokyo Shibaura Zoki. Measurements were performed within 36 h after slaughter. The normal ureter was cut into 1 cm cubes and the surrounding fatty tissue was separated using a scalpel. The remaining procedures were similar to those for human samples. All measurements were performed at room temperature.

### 2.2 Absorption and Reduced Scattering Coefficients

**Fig. 2:**
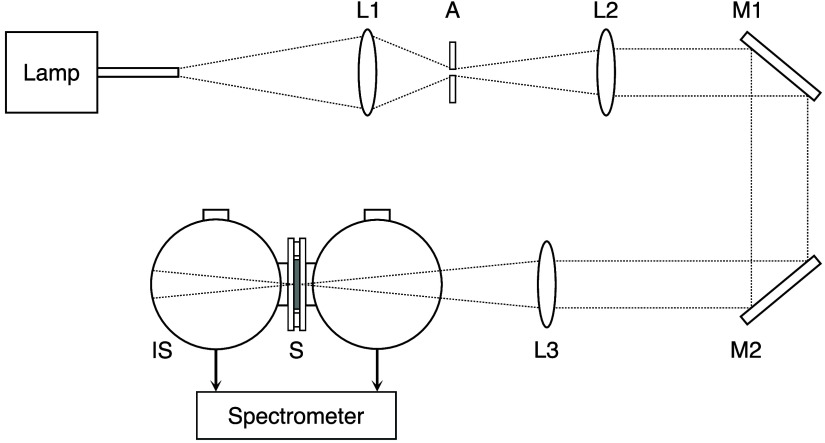
Schematic of the double integrating sphere optical setup. A, aperture; IS, integrating sphere; L1, L2, L3, lenses; M1, M2, mirrors; S, sample.

The $\mu_{a}$ and $\mu_{s}^{\prime }$ spectra were determined using a DIS optical system and IMC calculations.^17^ Figure 2 shows a schematic of the DIS optical system. A xenon lamp (XEF152-S, Kenko Tokina, Tokyo, Japan) was used as the light source. The light spectrum is shown in Fig. S1 in the Supplementary Material. The white light emitted from the light guide was focused with a condenser lens (f=32  mm, ACL50832U, Thorlabs, Newton, New Jersey, United States) through an iris diaphragm (ϕ=1  mm, IH-15R, OptoSigma, Tokyo, Japan), and then collimated (f=150  mm, AC254-150-AB, Thorlabs). After being reflected by mirrors (PF20-03-P01, Thorlabs), the light was focused (f=200  mm, AC254-200-AB, Thorlabs) onto the sample. The focused beam diameter remained within 1 mm before and after propagating through the sample along the optical axis, thus approximating a collimated beam. The reflected and transmitted light from the sample were both detected with a spectrometer (Maya2000Pro, Ocean Insight, Orlando, Florida, United States) via a DIS (4P-GPS-033-SL, Labsphere, North Sutton, New Hampshire, United States) and an optical fiber (P600-1-VIS-NIR, Ocean Insight). An adapter (PR-100-0250-SF, Labsphere) was used to make the port size 0.25 inch; therefore, the sample size was larger than the port size. A diffuse-reflectance standard (SRS-99-010, Labsphere) or a beam trap (BT610/M, Thorlabs) was used to measure the background of the $R_{d}$ spectrum. Exposure time ranged from 70 to 100 ms, which was adjusted based on the detected light intensity and fixed for each sample. Each measurement was averaged over 100 scans in a dark room and performed three times per sample. The DIS optical system was calibrated using diffuse-reflectance standards (SRS-10-010, SRS-20-010, Labsphere) and a transmittance filter (JCRM130, Japan Quality Assurance Organization, Tokyo, Japan). Differences between measured and calibrated values of $R_{d}$ and $T_{t}$ were within 0.8%. The $\mu_{a}$ and $\mu_{s}^{\prime }$ values were obtained from measured $R_{d}$ and $T_{t}$ using IMC calculations based on CUDAMCML.^17^^,^^18^ The refractive index (n) of the samples was set to be 1.4 by referring to literature values for kidney^19^ and bladder^20^ due to the lack of ones for the ureter. The anisotropy factor (g) of the samples was fixed at 0.9 because this value is typical for many tissues in the visible spectral range.^21^ The refractive index and the thickness of the glass slides were 1.524 and 1.0 mm, respectively. The average of three measurements was used as the measured value.

### 2.3 Calculation of Projected Light Penetration Depth

The δ values for each human and porcine tissue were calculated. When $\mu_{a}\ll 3\mu_{s}^{\prime }$, the projected light penetration depth can be written as^22^

$$\delta (\lambda )=\frac{1}{\sqrt{3\mu_{a}(\lambda )\{\mu_{a}(\lambda )+\mu_{s}^{\prime }(\lambda )\}}},$$ (1)

where λ is the wavelength. The δ values were compared between normal and cancerous tissues and between human and porcine tissues.

## 3 Results

### 3.1 Sample Thickness

The ureter sample thickness was compared between humans and pigs. The human and porcine ureter sample thicknesses were 1.2±0.4 and 0.6±0.2  mm, respectively. The porcine ureter thickness was less than that of the human ureter in the measured samples. The sliced thicknesses of human fatty tissue, ureteral carcinoma, and renal pelvic carcinoma were 1.5±0.3, 1.8±0.5, and 1.7±0.2  mm, respectively. The sliced porcine fatty tissue thickness was 1.6±0.2  mm.

### 3.2 Absorption and Reduced Scattering Coefficients

**Fig. 3:**
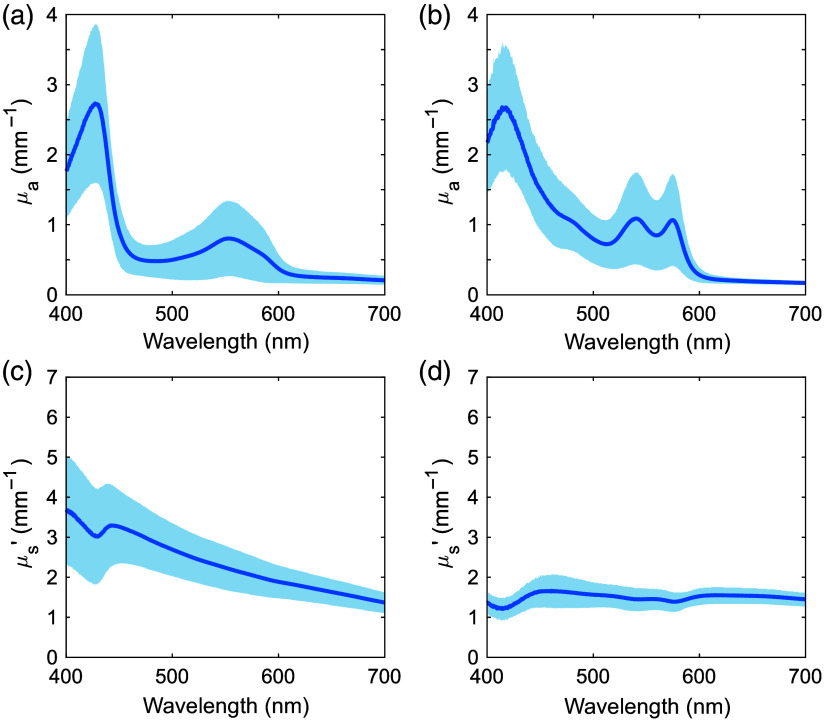
Absorption and reduced scattering coefficient spectra of human [(a), (c)] ureter and [(b), (d)] fatty tissue. The shaded areas represent standard deviations.

**Fig. 4:**
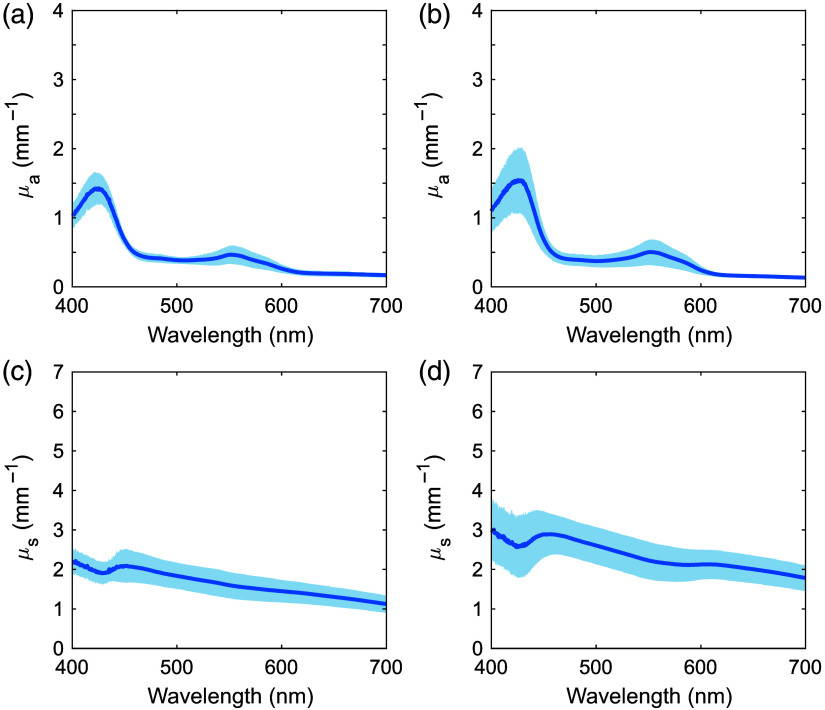
Absorption and reduced scattering coefficient spectra of human [(a), (c)] ureteral carcinoma and [(b), (d)] renal pelvic carcinoma. The shaded areas represent standard deviations.

Figures 3 and 4 show the $\mu_{a}$ and $\mu_{s}^{\prime }$ spectra for human ureter, fatty tissue, ureteral carcinoma, and renal pelvic carcinoma samples over the 400 to 700 nm range. The shaded regions show standard deviation. The sample numbers were 20 for the ureter, 15 for fatty tissue, five for ureteral carcinoma, and seven for renal pelvic carcinoma. In the $\mu_{a}$ spectrum of the ureter, primarily deoxygenated hemoglobin absorption peaks were observed at 427 and 550 nm.^23^ Similar trends were observed in the $\mu_{a}$ spectra of cancerous tissues. In the $\mu_{a}$ spectrum of fatty tissue, primarily oxygenated hemoglobin absorption peaks were observed at 416, 540, and 575 nm.^23^ Compared with fatty tissue, the ureter had slightly higher $\mu_{a}$ above 600 nm. In addition, a weak absorption peak due to bilirubin was observed at 485 nm.^24^ The $\mu_{s}^{\prime }$ spectra of all the samples monotonically decreased with increasing wavelength, which was attributed to the reduced contribution of Rayleigh scattering and the increased contribution of Mie scattering.^25^ The rate of decrease with wavelength was greatest in the ureter, followed by cancerous and fatty tissues.

**Fig. 5:**
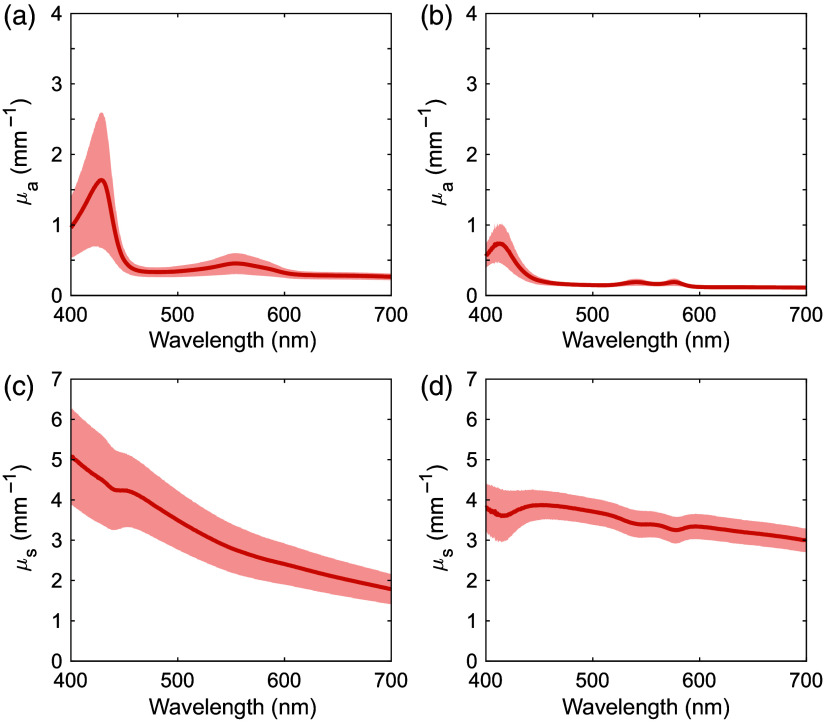
Absorption and reduced scattering coefficient spectra of porcine (a), (c) ureter and (b), (d) fatty tissue. The shaded areas represent standard deviations.

Figure 5 shows the $\mu_{a}$ and $\mu_{s}^{\prime }$ for porcine ureter and fatty tissue over 400 to 700 nm. The shaded regions show standard deviation. The information on sex was not provided. The sample numbers were nine for the ureter and nine for fatty tissue. In the $\mu_{a}$ spectrum of the ureter, primarily deoxygenated hemoglobin absorption peaks were observed at 427 and 550 nm, similar to human tissue.^23^ In the $\mu_{a}$ spectrum of fatty tissue, primarily oxygenated hemoglobin absorption peaks were observed at 412, 540, and 575 nm.^23^ The $\mu_{s}^{\prime }$ spectra of all tissues monotonically decreased with increasing wavelength. Table 1 summarizes the $\mu_{a}$ and $\mu_{s}^{\prime }$ values for each tissue for wavelengths used in PDT and LA.^26^^,^^27^

**Table 1:** List of absorption and reduced scattering coefficients of upper urinary tract tissues at wavelengths of 410, 510, 545, 580, 630, 635, 664, and 690 nm available for photodynamic therapy and 445, 450, and 532 nm for laser ablation.

Tissue type	Wavelength (nm)	Human		Pig	
		$\mu_{a}$ (${\mathrm{mm}}^{-1}$)	$\mu_{s}^{\prime }$ (${\mathrm{mm}}^{-1}$)	$\mu_{a}$ (${\mathrm{mm}}^{-1}$)	$\mu_{s}^{\prime }$ (${\mathrm{mm}}^{-1}$)
Ureter	410	2.16 ± 0.84	3.47 ± 1.33	1.19 ± 0.57	4.88 ± 1.16
	445	1.26 ± 0.56	3.29 ± 0.98	0.68 ± 0.27	4.24 ± 0.96
	450	0.90 ± 0.45	3.26 ± 0.91	0.50 ± 0.16	4.23 ± 0.92
	510	0.53 ± 0.32	2.58 ± 0.63	0.36 ± 0.09	3.34 ± 0.70
	532	0.65 ± 0.44	2.38 ± 0.58	0.40 ± 0.11	3.02 ± 0.64
	545	0.77 ± 0.51	2.28 ± 0.56	0.44 ± 0.14	2.86 ± 0.62
	580	0.62 ± 0.43	2.02 ± 0.47	0.39 ± 0.11	2.54 ± 0.54
	630	0.25 ± 0.09	1.74 ± 0.36	0.29 ± 0.05	2.20 ± 0.46
	635	0.25 ± 0.09	1.71 ± 0.35	0.29 ± 0.05	2.17 ± 0.46
	664	0.23 ± 0.08	1.56 ± 0.31	0.28 ± 0.05	1.99 ± 0.42
	690	0.21 ± 0.07	1.42 ± 0.27	0.27 ± 0.05	1.84 ± 0.39
Fatty tissue	410	2.53 ± 0.83	1.22 ± 0.30	0.72 ± 0.26	3.65 ± 0.64
	445	1.66 ± 0.62	1.64 ± 0.43	0.24 ± 0.06	3.87 ± 0.38
	450	1.51 ± 0.58	1.66 ± 0.43	0.21 ± 0.05	3.87 ± 0.36
	510	0.73 ± 0.34	1.56 ± 0.33	0.15 ± 0.02	3.66 ± 0.32
	532	1.00 ± 0.59	1.49 ± 0.34	0.18 ± 0.04	3.47 ± 0.34
	545	1.05 ± 0.64	1.46 ± 0.32	0.19 ± 0.05	3.39 ± 0.33
	580	0.93 ± 0.57	1.41 ± 0.27	0.18 ± 0.05	3.26 ± 0.32
	630	0.20 ± 0.05	1.55 ± 0.24	0.12 ± 0.02	3.24 ± 0.30
	635	0.20 ± 0.04	1.55 ± 0.23	0.12 ± 0.02	3.22 ± 0.30
	664	0.18 ± 0.03	1.53 ± 0.22	0.11 ± 0.02	3.13 ± 0.30
	690	0.17 ± 0.03	1.48 ± 0.20	0.11 ± 0.02	3.04 ± 0.29
Ureteral carcinoma	410	1.23 ± 0.20	2.10 ± 0.32	—	—
	445	0.85 ± 0.13	2.06 ± 0.37	—	—
	450	0.68 ± 0.09	2.09 ± 0.40	—	—
	510	0.38 ± 0.05	1.78 ± 0.30	—	—
	532	0.40 ± 0.09	1.69 ± 0.31	—	—
	545	0.45 ± 0.12	1.63 ± 0.31	—	—
	580	0.36 ± 0.10	1.50 ± 0.28	—	—
	630	0.20 ± 0.04	1.37 ± 0.23	—	—
	635	0.19 ± 0.04	1.35 ± 0.23	—	—
	664	0.19 ± 0.03	1.26 ± 0.22	—	—
	690	0.17 ± 0.03	1.16 ± 0.20	—	—
Renal pelvic carcinoma	410	1.33 ± 0.37	2.80 ± 0.74	—	—
	445	0.89 ± 0.27	2.85 ± 0.65	—	—
	450	0.69 ± 0.18	2.88 ± 0.58	—	—
	510	0.38 ± 0.10	2.53 ± 0.46	—	—
	532	0.42 ± 0.13	2.36 ± 0.46	—	—
	545	0.48 ± 0.17	2.26 ± 0.47	—	—
	580	0.38 ± 0.13	2.12 ± 0.41	—	—
	630	0.17 ± 0.03	2.07 ± 0.34	—	—
	635	0.16 ± 0.02	2.06 ± 0.34	—	—
	664	0.15 ± 0.02	1.95 ± 0.33	—	—
	690	0.14 ± 0.02	1.83 ± 0.32	—	—

### 3.3 Projected Light Penetration Depths of Normal and Cancerous Human Tissues

**Fig. 6:**
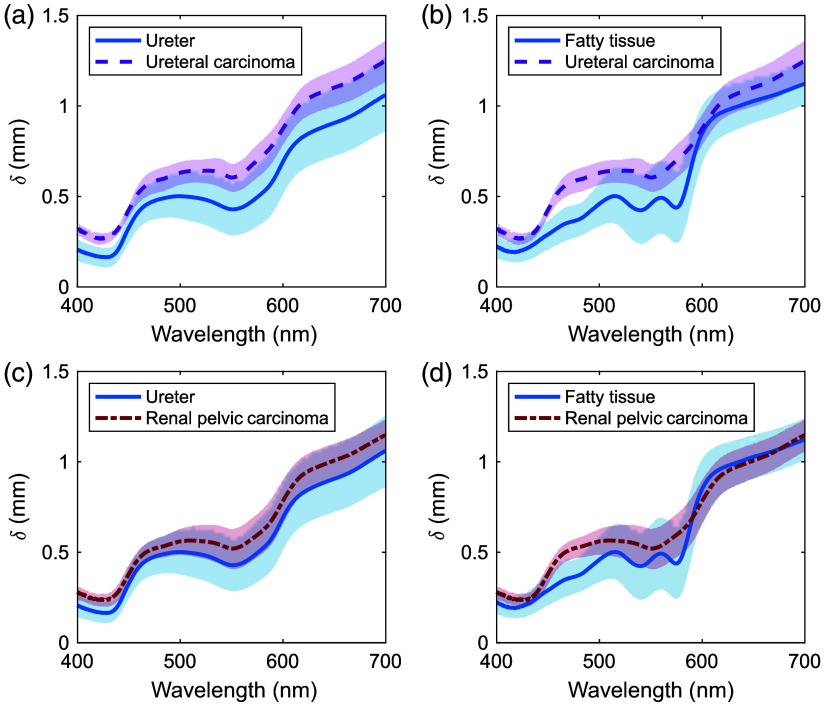
Comparison of projected light penetration depths between (a) human ureter and ureteral carcinoma, (b) human fatty tissue and ureteral carcinoma, (c) human ureter and renal pelvic carcinoma, and (d) human fatty tissue and renal pelvic carcinoma.

Figures 6(a) and 6(b) compare δ values of the ureter and fatty tissues with those of the ureteral carcinoma. The shaded regions show standard deviation. The δ values of the ureter and ureteral carcinomas exhibited approximately the same trends with increasing wavelength, where the tumor values were 1.2 to 1.7 times those of the ureter. The difference in δ between the fatty tissue and ureteral carcinoma was pronounced in the 450 to 600 nm range because of the primarily oxygenated hemoglobin absorption peak. At 600 nm, there was almost no difference between fatty tissue and ureteral carcinoma. Figures 6(c) and 6(d) compare the δ values of the ureter and fatty tissue with that of the renal pelvic carcinoma. Similar to the ureteral carcinoma, the δs for the renal pelvic carcinoma showed roughly the same trend with increasing wavelength, with the tumor values being 1.1 to 1.5 times those of the ureter. The δs of fatty tissue and renal pelvic carcinoma showed almost no differences at 425 and above 600 nm.

### 3.4 Projected Light Penetration Depths of Normal Human and Porcine Tissues

**Fig. 7:**
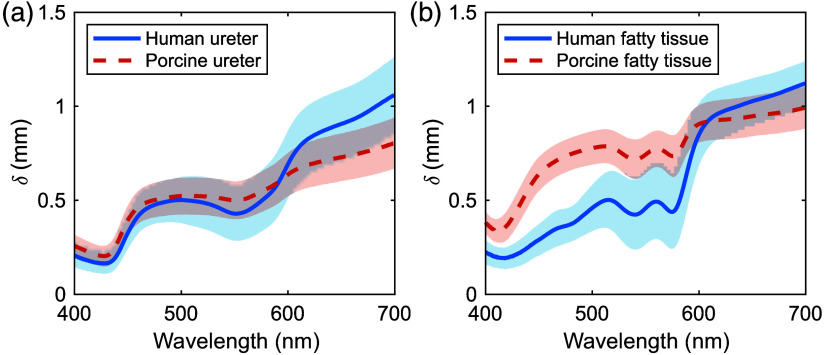
Comparison of projected light penetration depths between (a) human and porcine ureters and (b) human and porcine fatty tissues.

Figures 7(a) and 7(b) compare δ values of human and porcine ureters and fatty tissues, respectively. The δ values of the porcine ureter were similar to those of the human ureter below 600 nm but were up to 0.2 times less above 600 nm. The δ values of porcine fatty tissue were up to 1.2 times greater than those of human tissue below 600 nm but up to 0.1 times less above 600 nm.

### 3.5 Sensitivity Analysis of g and n

**Fig. 8:**
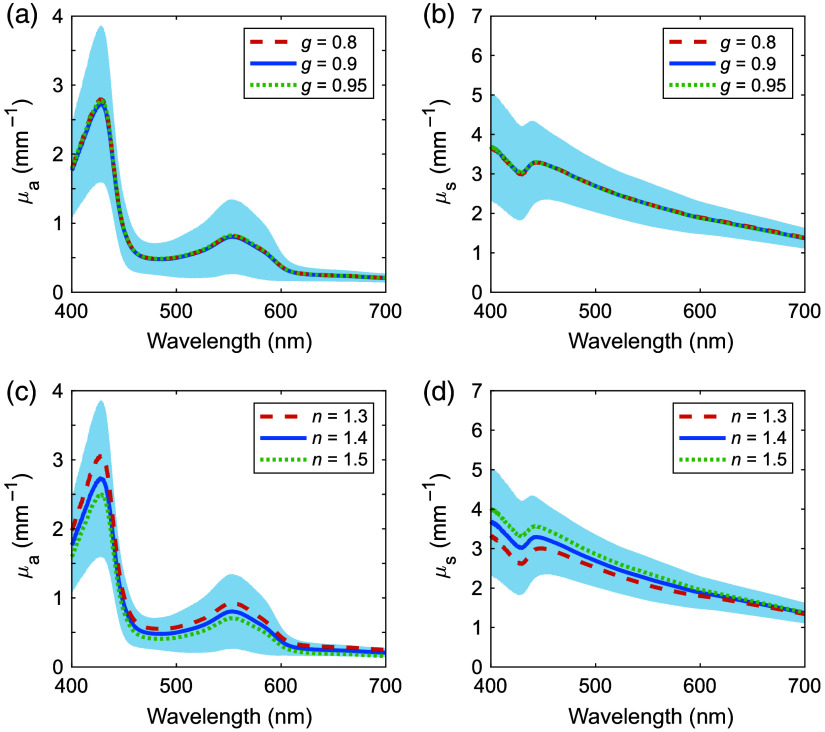
(a) Absorption; (b) reduced scattering coefficient spectra of human ureter obtained for g=0.8, 0.9, 0.95, and n=1.4; (c) absorption; and (d) reduced scattering coefficient spectra of human ureter obtained for g=0.9 and n=1.3, 1.4, and 1.5. The shaded areas represent standard deviations for g=0.9 and n=1.4.

In the IMC calculations, g and n were assumed to be 0.9 and 1.4, respectively. To investigate the effects of g and n on the measured $\mu_{a}$ and $\mu_{s}^{\prime }$ spectra, sensitivity analyses were performed. g values were varied over 0.8, 0.9, and 0.95 by reference to previous work on the brain and skin,^17^^,^^22^^,^^28^ and n values were varied over 1.3, 1.4, and 1.5 by reference to a literature on the refractive indices of the kidney, liver, stomach, colon, and esophagus.^29^ Figures 8(a) and 8(b) show the $\mu_{a}$ and $\mu_{s}^{\prime }$ spectra of human ureter, respectively, with n fixed at 1.4 and varying g, whereas Figs. 8(c) and 8(d) show the $\mu_{a}$ and $\mu_{s}^{\prime }$ spectra, respectively, with g fixed at 0.9 and varying n. When g was varied, there were almost no changes in $\mu_{a}$ and $\mu_{s}^{\prime }$. However, when n was varied, $\mu_{a}$ had variations of ±21% when n=1.3 and −24% when n=1.5, and $\mu_{s}^{\prime }$ exhibited variations of ±10% when n=1.5 and −13% when n=1.3.

## 4 Discussion

The $\mu_{a}$ and $\mu_{s}^{\prime }$ spectra of human ureter, fatty tissue, ureteral carcinoma, and renal pelvic carcinoma, and porcine ureter and fatty tissue samples were acquired in the visible wavelength range. The variations in $R_{d}$ and $T_{t}$ values were greater than 10% of the average. These variations were larger than the measurement accuracy of the DIS optical system and therefore attributed to individual differences in the tissue samples. In the $\mu_{a}$ spectra of human ureter and fatty tissue, the standard deviations were large in the 400 to 600 nm range. This could be attributed to differences in the amount of oxygenated and deoxygenated hemoglobins in the samples. The $\mu_{a}$ spectrum of human fatty tissue also showed large standard deviations in the 450 to 500 nm range. This was likely due to the bilirubin absorption peak, indicating variability in bilirubin content in the samples.^24^ In addition, the effects of oxygenated and deoxygenated hemoglobins decreased in the human ureter and fatty tissues above 600 nm due to the absence of absorption peaks, resulting in less variation. Although the $\mu_{a}$ of oxygenated and deoxygenated hemoglobins at this wavelength range were less than those at their peak wavelengths, deoxygenated hemoglobin absorbs more light than oxygenated hemoglobin at 600 to 810 nm.^23^ This difference may be the reason why the $\mu_{a}$ values of human ureter, which was more affected by deoxygenated hemoglobin, was slightly higher than those of human fatty tissue.

The $\mu_{s}^{\prime }$ values had different spectra among the different tissue types. Human and porcine ureters consisted of mucosal epithelium, connective tissue, and muscle tissue. Human and porcine fatty tissue comprised mainly adipocytes. Cancerous tissues composed mainly of malignant epithelial tissue. These different compositions may be responsible for the differences in $\mu_{s}^{\prime }$ among the different tissue types.^17^^,^^30^ The differences in $\mu_{s}^{\prime }$ between human and porcine tissues may be attributed to the different density of the scatterers, including cells, collagen, and elastin.^31^ At wavelengths below 480 nm, where Rayleigh scattering is sensitive,^32^ a steeper slope of the $\mu_{s}^{\prime }$ spectra with higher values was observed for the ureter relative to that for the fatty tissue for both human and porcine samples. This suggests that the Rayleigh scattering contribution may have been more pronounced in the ureter.^9^ In fatty tissue, adipocytes contain lipid droplets that are larger than other intracellular components.^33^^,^^34^ Therefore, the Mie scattering contribution to the $\mu_{s}^{\prime }$ spectra of the fatty tissue increased, and consequently, the Rayleigh scattering contribution would be expected to decrease.

The variations in $\mu_{a}$ and $\mu_{s}^{\prime }$ observed when g was varied were less than the standard deviations of $\mu_{a}$ and $\mu_{s}^{\prime }$ for g=0.9, indicating that the measurement of $\mu_{a}$ and $\mu_{s}^{\prime }$ were insensitive to g. This result was consistent with IMC-based optical property analysis of the human skin and brain.^17^^,^^22^^,^^28^ When n was varied, the changes in $\mu_{a}$ and $\mu_{s}^{\prime }$ were within the range of their standard deviations; however, $\mu_{a}$ changed by ∼20%, and $\mu_{s}^{\prime }$ by ∼10%. The variations in optical properties attributed to the refractive index must be considered when evaluating laser treatments. Numerous optical techniques have been developed to quantitatively assess the refractive index of biological samples,^29^ and for future work, refractive index measurements would be helpful in improving the accuracy of the IMC calculations.

In the measured values, the oxidation–reduction states of hemoglobin differed between the ureter and fatty tissues, which was possibly effected by sample preparation. The ureter contained a network of capillaries,^10^ making it less susceptible to oxidation after sample extraction. By contrast, fatty tissue had large blood vessels,^10^ and when the sample was cut, the exposed blood easily came into contact with air, facilitating oxidation. This suggests that the hemoglobin oxidation–reduction state and blood volume may differ from *in vivo* conditions, and better control of the oxygenation conditions of the samples is needed. The samples were preserved in saline moistened gauze to reduce ambient oxygenation and sample desiccation in this experiment. In addition, the metabolic activity of the cells can be reduced by storing the samples in glass vials and cooling them until before measurement, reducing oxygen consumption.^35^ This may allow better control of the oxygenation conditions of the samples more than if they were stored at room temperature. To study samples under different oxygenation conditions, a method of measuring samples over time could be used. This allows evaluation of changes in the hemoglibin oxidation–reduction state that occur during measurement. In addition, because the $\mu_{a}$ of the tissue can be determined by summing the $\mu_{a}$ of chromophores weighted by volume fraction,^9^ optical properties of the tissue under different oxygenation conditions can be analyzed by measuring samples from which the blood has been removed and then correcting the measured values for the amount of oxygenated and deoxygenated hemoglobins.

Ureter samples used here consisted of mucosal epithelium, connective tissue, and muscle tissue. Therefore, $\mu_{a}$ and $\mu_{s}^{\prime }$ spectra could be regarded as optical properties averaged over volume fractions of each tissue. The optical properties of solely mucosal and muscle tissues may differ from those measured here. *In vivo* techniques for measuring optical properties, such as spatial frequency domain imaging^36^ and diffuse reflectance spectroscopy,^37^ have been proposed. However, because of limitations in the size of the optical system and the number of fiber optic patch cables, it was difficult to access narrow regions within the body, such as the urinary tract, to obtain spectral information. In addition, to ensure the accuracy of cancerous tissue measurements, it is necessary to account for inhomogeneous tissue structures in the inverse problem analysis. To overcome these limitations, this experiment used *ex vivo* a DIS optical system. By slicing the samples to ∼1  mm thicknesses, the system enabled acquisition of optical properties for both normal and cancerous tissues and a simple inverse problem analysis. The DIS system could simultaneously measure $R_{d}$ and $T_{t}$, which reduced the measurement time and thus minimized sample degradation. Moreover, the IMC calculations can provide more accurate $\mu_{a}$ and $\mu_{s}^{\prime }$ values for a wide range of measured values than the inverse adding–doubling method.^15^

δs are affected by both optical absorption and scattering. The $\mu_{a}$ and $\mu_{s}^{\prime }$ values of human fatty tissue were similar to those of ureteral carcinoma at 600 nm. This relationship in optical properties resulted in the lack of differences in δ between fatty tissue and ureter carcinoma at this wavelength [Fig. 6(b)]. At 430 nm, the $\mu_{a}$ value of the human fatty tissue was greater than that of renal pelvic carcinoma, whereas the $\mu_{s}^{\prime }$ value was less for human fatty tissue, resulting in the absence of differences in δ between fatty tissue and renal pelvic carcinoma at this wavelength [Fig. 6(d)].

The δs were calculated using the analytical expression (Eq. 1) to compare light penetrability into the tissue between different wavelengths. This analytical expression can be used to simply estimate the differences in light distribution in the tissue between wavelengths as opposed to Monte Carlo (MC) simulations that require modeling the optical structure of the ureter, fat, and cancerous tissue. However, the extent to which the relationship of $\mu_{a}\ll 3\mu_{s}^{\prime }$ was satisfied by the measured values, especially for human fatty tissue, decreased with decreasing wavelength. Therefore, it is necessary to calculate the light distribution in the tissue using MC simulation to obtain accurate penetration depths.

Because δ is a function of wavelength, evaluating whether it covers the target depth can help narrow candidate wavelengths for treatment. In addition, selected wavelengths should be absorbed by endogenous or exogenous chromophores within the target. Comparing candidate wavelengths from this perspective also narrows the treatment wavelengths. In PDT, the absorption peaks of the photosensitizer are candidate wavelengths. In this experiment, the δ was greater in ureteral and renal pelvic carcinomas relative to that in normal tissues. This result indicated that if the irradiation parameters were selected by considering only normal tissues, the dose may be excessive. Therefore, it is necessary to consider differences in optical properties between normal and cancerous tissues when setting irradiation parameters for treating UTUC.

By comparing the $\mu_{a}$ and $\mu_{s}^{\prime }$ spectra and δs for each human and porcine tissue, valuable information can be obtained regarding the validity of using porcine tissues as an *ex vivo* model, as well as differences when extrapolating porcine results to human tissues. The comparison of δs between porcine and human ureter tissues indicated nearly identical values in the 400 to 600 nm range; the depth was ∼0.2 times smaller in porcine tissues above 600 nm. When evaluating treatments using 400 to 600 nm light, the porcine ureter may serve as an *ex vivo* model comparable with the human ureter. However, for treatments using light greater than 600 nm, data obtained from the porcine ureter may underestimate the depth of light interactions with the human ureter, potentially affecting the evaluation of treatment effects. When comparing human and porcine fatty tissues, the δ was up to 1.2 times greater below 600 nm for porcine fatty tissue due to the optical absorption by primarily oxygenated hemoglobin. Above 600 nm, the δ was up to 0.1 times less for porcine fatty tissue due to the greater effect of scattering. Data obtained from the porcine fatty tissue may overestimate the effect of light on the human fatty tissue in the depth direction below 600 nm, and they may underestimate the effect of light on the human fatty tissue in the depth direction above 600 nm.

Tissue structure should also be considered when evaluating the validity of extrapolating data obtained from the porcine tissue to the human tissue because light distributions in the tissue depend not only on optical properties but also on tissue structure. The average sample thickness of the porcine ureter was ∼0.6  mm thinner than that for the human ureter samples. This fact is a drawback when using porcine models for the analysis of laser treatment. Hence, thickness differences must be considered when extrapolating data from porcine to human tissue. The thicknesses of the human and porcine ureters were 1.2±0.4 and 0.6±0.2  mm, respectively, whereas the δs at 635 nm available for PDT were 0.9±0.2 and 0.7±0.1  mm, respectively.^26^ The human ureter thickness covered the δ, whereas the porcine ureter thickness did not. This difference suggests that the use of porcine ureter may be inconclusive to evaluate PDT. However, light at 450 nm and 532 nm penetrated to depths of ∼0.5  mm or less, which was less than the thicknesses of the human and porcine ureter samples. This suggests that the porcine ureter may serve as an optical model for preclinical evaluation of LA at these wavelengths.^27^ However, the δs are indicators of laser treatment depth that only consider wavelength. The actual treatment depth is affected by multiple factors, including the shape of the light source, irradiation power, irradiation time, beam profile, beam divergence, tissue shape, and layer structures. To evaluate the impacts of these factors on treatments, the light distributions in the tissues must be analyzed via numerical modeling of the upper urinary tract and MC simulations of light transport.

## 5 Conclusion

We measured the $\mu_{a}$ and $\mu_{s}^{\prime }$ spectra for normal and cancerous human tissues, as well as normal porcine upper urinary tract tissues, over the 400 to 700 nm range using a DIS optical system and IMC calculations. The measured values were used to calculate δ in each tissue for comparative evaluations between normal and cancerous tissues and between human and porcine tissues. The δ values were greater in ureteral and renal pelvic carcinomas relative to those in normal human tissues, suggesting that differences in optical properties between normal and cancerous human tissues should be considered when setting laser irradiation parameters. In addition, a comparison of δs of human and porcine ureteral tissues showed almost no differences below 600 nm, but porcine tissues were up to 0.2 times smaller above 600 nm. For the 400 to 600 nm range, the porcine ureter could thus serve as an *ex vivo* model for the human ureter in terms of optical properties. However, for wavelengths longer than 600 nm, data obtained from porcine tissues may underestimate the depth of light effects in human tissues. These experimental results are expected to provide valuable information on light distributions in the human upper urinary tract for establishing effective *ex vivo* tests for light-based therapeutic approaches for UTUC. Future studies will need to analyze the light distribution in the upper urinary tract tissue using light propagation simulations to evaluate detailed irradiation conditions for treatments.

## Supplementary Material



## Data Availability

The datasets used and/or analyzed during the current study are available from the corresponding author on reasonable request.
