# Supplementary material for: Measurement of tissue optical properties in the 400 to 700 nm range to assess light penetration depths for laser treatment of upper tract urothelial carcinomas
Source: J Biomed Opt. 2024 Dec 10;29(12):125001. doi: 10.1117/1.JBO.29.12.125001 (PMC11629118; doi:10.1117/1.JBO.29.12.125001)
Supplement: Supplementary file 1 [file JBO_029_125001_SD001.docx]

**Measurement of tissue optical properties in the 400--700 nm range to assess light penetration depths for laser treatment of upper tract urothelial carcinomas**

**Supplementary information**

Himemi Watabe^a,†^, Yu Shimojo^a,b,c,†,*^, Asako Shingu^d^, Hidenori Ito^d^, Hideo Fukuhara^e^, Makito Miyake^f^, Keiji Inoue^e^, Kiyohide Fujimoto^f^, Takahiro Nishimura^a,*^

^a^Osaka University, Graduate School of Engineering, Osaka, Japan

^b^Osaka Metropolitan University, Graduate School of Medicine, Osaka, Japan

^c^Research Fellow of Japan Society for the Promotion of Science, Tokyo, Japan

^d^SBI Pharma CO., LTD., Tokyo, Japan

^e^Kochi Medical School, Kochi University, Department of Urology, Kochi, Japan

^f^Nara Medical University, Department of Urology, Nara, Japan

†These authors contributed equally to this work.

*Address all correspondence to Yu Shimojo, E-mail: x22800k@omu.ac.jp and Takahiro Nishimura, nishimura-t@see.eng.osaka-u.ac.jp

**
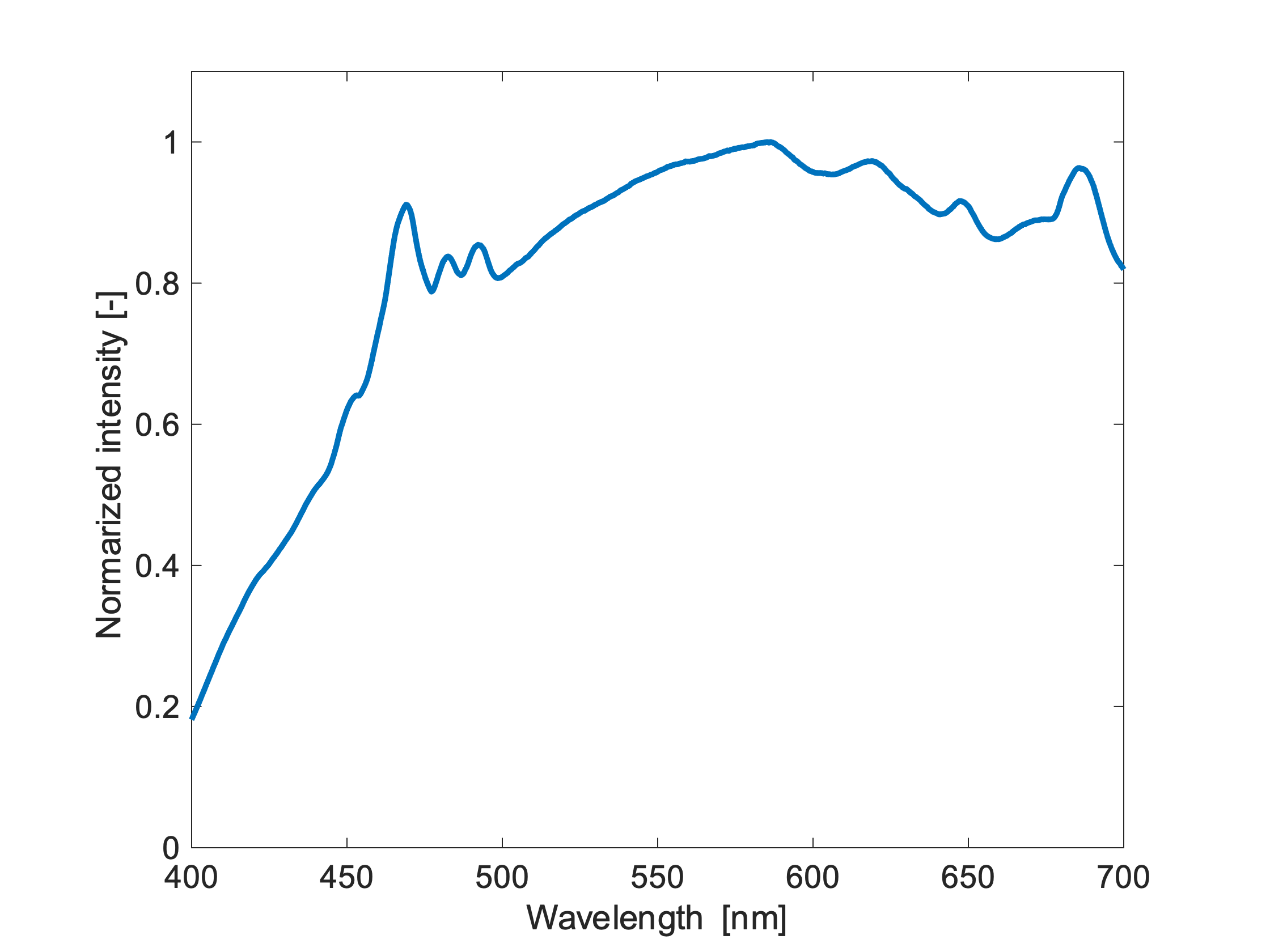
**

**Fig. S1** Light spectrum of a xenon lamp used in the double integrating sphere optical system.
